# Protein Target Quantification Decision Tree

**DOI:** 10.1155/2013/701247

**Published:** 2013-01-15

**Authors:** Jong Won Kim, Jinsam You

**Affiliations:** Department of Biochemistry and Molecular Biology, Indiana University School of Medicine, 635 Barnhill Drive, MS 4053, Indianapolis, IN 46202, USA

## Abstract

The utility of mass spectrometry-(MS-) based proteomic platforms and their clinical applications have become an emerging field in proteomics in recent years. Owing to its selectivity and sensitivity, MS has become a key technological platform in proteomic research. Using this platform, a large number of potential biomarker candidates for specific diseases have been reported. However, due to lack of validation, none has been approved for use in clinical settings by the Food and Drug Administration (FDA). Successful candidate verification and validation will facilitate the development of potential biomarkers, leading to better strategies for disease diagnostics, prognostics, and treatment. With the recent new developments in mass spectrometers, high sensitivity, high resolution, and high mass accuracy can be achieved. This greatly enhances the capabilities of protein biomarker validation. In this paper, we describe and discuss recent developments and applications of targeted proteomics methods for biomarker validation.

## 1. Introduction 

Recently, advanced proteomics technology and instrumentations has allowed for the generation of more than a thousand candidate biomarkers from the profiling of complex biological samples. Most of these proteins were from under powered studies or pooled samples that had a large number of hypotheses being tested in similar conditions. Protein biomarkers have great potential to improve diagnosis, guide targeted therapy, and monitor therapeutic response across a wide range of diseases [1]. Mass spectrometry-based proteomics has become a powerful tool for biomarker discovery and validation in recent years [2–4]. However, to date, no protein biomarker identified using proteomics has been introduced into clinical use [5–9]. Although “omics” technologies have revolutionized the discovery of candidate biomarkers, several major technological limitations, including sensitivity, accuracy, and reproducibility, have hindered the application of proteomics as a platform for biomarker research. Discovery proteomics has enabled the identification of hundreds of biomarker candidates in many disease types, but the lack of well-established methods for validation of the biomarker candidates involving a large number of clinical samples is blamed for the low yield of clinically useful biomarkers [10–12]. The linkage between new technological platforms and the discovery of truly disease-related biomarkers needs to be established before moving candidate protein biomarkers toward clinical implementation. Recent advances in mass spectrometry and bioinformatics now enable construction of a comprehensive biomarker pipeline from six essential process components: candidate discovery, qualification, verification, assay development and optimization, candidate validation, and commercialization.

Targeted proteomics has emerged as a promising high-throughput platform for biomarker candidate validation, as well as systems biology applications. Centered on selected reaction monitoring (SRM) mass spectrometry, quantitative targeted proteomics has been used in the verification and validation of discovery data. SRM or Multiple Reaction Monitoring (MRM) is a target quantification technology with greatest selectivity (specificity) routinely performed on either a triple-quad or an ion-trap mass spectrometry. It has been widely used in small molecule quantification and research for decades [13]. It isolates a selected precursor ion in the first quadrupole (Q1), produces product ions by collision-induced dissociation (CID) in Q2, and filters one or multiple predefined product ions in Q3. The ion count of the product ion(s) in Q3 represents the amount of the targets. For the ion trap instrument, Q1 function in the triple-quad can be mimicked with maximum sensitivity by enabling injection waveforms in the tune file of the ion trap (e.g., LTQ). The target selection by two unique signatures from Q1 and Q3 and chromatographic separation create a great selectivity nature [14]. SRM technical details and target peptide/protein quantification guidelines are well documented [15, 16].

Many biomarker discovery studies have been performed using human biological fluids, because it is relatively easy to access and has a high potential for application to clinical research. High abundant protein removal and multiple target enrichment techniques were employed to achieve low abundant biomarker candidate quantification. Without additional sample enrichment or fractionation, most advanced triple-quad or ion-trap mass spectrometry alone offer a limit of quantitation (LOQ) down to the high ng/mL range; however, many clinically important biomarkers are in the low ng/mL range in the blood. Since sensitivity is one of the challenges for SRM-based assays, lots of efforts have been focused on hardware development and target enrichment techniques to improve the SRM assay sensitivity. Field asymmetric ion mobility spectrometry (FAIMS) increased sensitivity via improving the signal-to-noise ratio, and it achieved 1 nM of standard peptide in rat plasma [17]. The combination of a multicapillary inlet and dual funnel ion channel technology reached 20- to 150-fold intensity improvement from regular SRM [18]. The multicapillary inlet transfers significantly more ions to the mass spectrometry, and the dual funnel ion channel captures, focuses, and transfers ions more efficiently to achieve high sensitivity [18]. SRM3 in hybrid mass spectrometry also lowered limit of detection (LOD) to 1.5 ng/mL in one application, and it still has potential to gain more sensitivity by capturing only one filtered ion with the trap [19]. 

**Figure 1:**
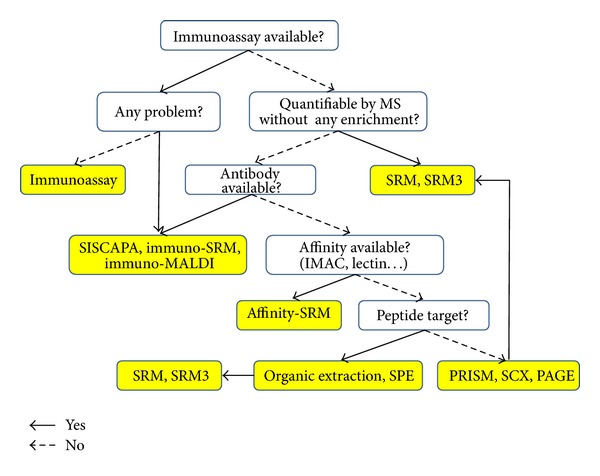
Protein target quantification decision tree.

Although many advanced technologies are available, none of the single technology platforms can cover all of the possible protein targets at once. This paper provides a decision tree (Figure 1) to choose the proper tools and technologies for protein/peptide target quantification to take advantage of each method. 

## 2. Are There Any Available Assays for the Target(s)?

Immunoassays have been used as the gold standard for decades to measure specific protein/peptide targets from serum/plasma, tissue, or proximal fluids [20]. If the validated antibody-based assay is available, it may still be the first option for target protein quantification due to its high sensitivity, high throughput, and cost effective nature. It typically requires a pair of well characterized antibodies, and most of the commercially available immunoassays follow the FDA's bioanalytical method development and validation guideline [21]. Almost 90 of the FDA-approved immunoassays are readily available, and the number is over 200 if all clinical protein tests are included [22]. Currently, they cover less than 1% of the total human proteome, but many research groups are actively developing new assays to meet important medical needs. While antibody-based assays most commonly use the monoplex assay format, multiplexed immunoassays have also been adopted [23–26]. There are planar assays and suspension microsphere assays. Only few multiplexed assays are currently approved by the FDA [27, 28]; however, other multiplexed immunoassays are also available for diagnostics and research purposes [29, 30].

**Figure 2:**
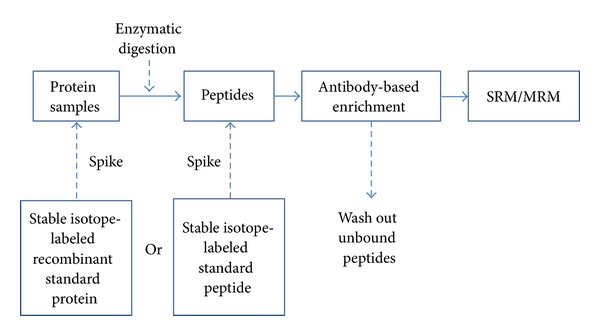
Schematic view of Stable Isotope Standards and Capture by Anti-Peptide Antibodies (SISCAPA).

Current immunoassay platforms have a certain intrinsic limitation because of the existence of interfering substances, such as autoantibodies of the target and close relatives, which negatively affects clinical performance [31, 32]. To overcome autoantibody interference, Anderson et al. implemented the Stable Isotope Standards and Capture by Anti-Peptide Antibodies (SISCAPA) technique (Figure 2) [33]. (this technique will be discussed further in a later section) in a clinical laboratory environment for the measurement of serum thyroglobulin [34]. To distinguish close relative species, Niederkofler et al. used mass spectrometry immunoassay (MSIA) tips and matrix-assisted laser desorption/ionisation-time of flight (MALDI-TOF) for B-type natriuretic peptide (BNP) measurements from heart failure patients [32]. They demonstrated a potential reason of the “natriuretic paradox.” Unlike MSIA, a commercially available immunoassay cannot distinguish active and inactive BNP, and it overestimates BNP's biological activity [32]. Lopez et al. used the mass spectrometric immunoassay (MSIA) tips and SRM for the quantification of parathyroid hormone and variants for the accurate diagnosis of endocrine disease and osteoporosis [35]. Advantages, challenges, and possible applications of each target quantification technique were briefly summarized in Table 1.

## 3. Quantifiable by Mass Spectrometry without Enrichments?

Since quantitative immunoassays are not available for most proteins [36] or impossible to be applied as immunoassay alone in certain cases, liquid chromatography-mass spectrometry-(LC-MS-) based quantification of biomolecules may be an attractive forward option. Blood has a very wide dynamic range of protein concentrations. Standard LC-SRM can generally detect proteins down to the low ug/mL range. Anderson and Hunter demonstrated that the top 47 proteins can be quantified from a single run without extensive sample enrichment using nano-LC-SRM [37]. Domanski et al. expanded this idea using a high flow ultra-high pressure liquid chromatography (UHPLC or UPLC) SRM and quantified 117 proteins from human plasma without depletion or enrichment, including 84 known to be cardiovascular disease biomarkers [38]. Many of these proteins are in the available immunoassay list. Since the SRM-based approach is multiplexed, it may be a better option when multiple targets have to be quantified. Since these approaches use plasma without depletion or enrichment, the assay can be higher throughput and less expensive. In recent years, almost all high-pressure liquid chromatography (HPLC) companies have released their own cutting-edge UPLC system to achieve higher speed (thus throughput), better resolution, and greater sensitivity. Higher speed can be attained by pumping at a higher solvent flow rate without affecting the resolution. Higher resolution can be achieved by using resin with a smaller particle size or by using a longer column. Although high-flow LC (2.1 mm, 400 uL/min) is less sensitive than nano LC (75 um, 300 nL/min), it improves retention time reproducibility and narrows peak width [38]. Since the plasma amount is not limited and more protein can be loaded on the larger column, the loss of sensitivity can be partially compromised, and it is potentially a very useful tool to triage high-to-moderate-abundant biomarker candidates from serum/plasma without a significant investment on reagents. 

Recently, selectivity has been improved even more by using a hybrid mass spectrometer. A triple-quadrupole/linear ion-trap hybrid instrument has a so-called MRM3 mode which uses the same Q1 precursor selection and Q2 fragmentation; however, it traps the most intense optimally defined product ion in Q3, generates a second generation of product ions by activation in the linear ion trap, and filters predefined second generation product ion(s) [19]. Although MRM3 has intrinsic limitations due to its slow duty cycle (300 milliseconds), it has great value in certain applications. Due to its greater selectivity, MRM3 should be able to quantify close related peptides from complex mixture with minimal sample preparation. 

## 4. Is There an Available Antibody for the Target?

Low abundant protein target quantification from plasma has been challenging because of the very wide dynamic of protein concentrations in plasma [39, 40]. As described in the previous section, advancements in mass spectrometry technology have allowed for the detection of plasma proteins in the low ng/mL range without enrichment [18, 19]. When target proteins are low in abundance and an immunoassay is not available or is problematic, immunoaffinity is likely the most efficient method for target enrichment due to its sensitivity and selectivity nature. Immunoaffinity coupled mass spectrometry-based assays using electrospray ionization (ESI) or MALDI have allowed for reliable target protein quantification from blood samples [4, 32, 35, 41, 42]. Berna et al. used immuno-SRM to measure one of the drug-induced cardiotoxicity markers from rat plasma after initial enzyme-linked immunosorbent assay (ELISA) development failed [41]. If the antibody is available for the target protein, SISCAPA [37, 43] may be another option, and it can even be multiplexed (Figure 2). This approach uses anti-peptide antibodies to enrich for the target tryptic signature peptides from the total tryptic digest. If a stable isotopically labeled recombinant standard protein is available, it can be spiked into each sample prior to trypsin treatment. Both heavy and light peptides are eluted from the immobilized antibody and quantified by the SRM technique. In this case, the secondary antibody function is replaced by mass spectrometry. By using magnetic beads to immobilize the antibody, the enrichment steps can be handled by robotics, and reproducibility and throughput will be significantly improved [43]. 

Immuno-SRM was also applied to quantify certain posttranslational modifications such as tyrosine-phosphorylation and lysine-acetylation [44, 45]. Wolf-Yadlin et al. used an anti-phosphotyrosine antibody and an immobilized metal affinity chromatography (IMAC) column as enrichment tools for phosphopeptide and quantified 222 phosphotyrosine-containing peptides after epidermal growth factor (EGF) stimulation [44]. Drogaris et al. reported quantification of histone lysine-acetylation after treatment of histone deacetylase inhibitors using immuno-SRM [45]. Unlike ELISA, these techniques do not require a pair of antibodies. A nonspecific antibody may also be used in the initial enrichment step if it has high affinity, because mass spectrometry has high resolving power. The best sensitivity comes from antibody enrichment, and a great selectivity comes from the SRM nature. Due to these reasons, SRM may now be recognized as an alternative technology to ELISA [12]. The marriage of these two excellent features may be the most powerful approach in biomarker research. 

## 5. Are There Other Affinity Techniques Available for the Target?

When targets are in low abundance and no antibody is available, other affinity enrichment technique coupled with SRM may need to be considered. Besides phosphorylation, glycosylation is another important posttranslational modification (PTM) that potential biomarker candidates may possess. Glycosylated protein targets were generally enriched by lectins [46] and hydrazide chemistry without an antibody [47]. Ahn et al. used a fucose-specific aleuria aurantia lectin coupled with SRM to identify biomarkers for liver cancer without antibody [46]. N-glycosylated proteins can be oxidized and captured with hydrazide resin, and the glycosylated peptides can be released by Peptide N-Glycosidase F (PNGase) treatment [47]. The eluted peptides can be measured by MALDI or ESI mass spectrometry [47, 48]. 

## 6. Peptide Target

There are many small peptide biomarker candidates in biological fluids such as blood, urine, and cerebrospinal fluid (CSF). To quantify the naturally existing peptides, the interfering substances should be eliminated from the complex matrix. Unlike large proteins, many peptides have a great solubility in organic solvent. Organic extraction precipitation or solid phase extraction (SPE) can be applied to enrich many small peptide targets [49]. By using a 96 well plate format, overall throughput can be increased. These techniques readily achieved detection in the single-digit ng/mL range from blood samples [50]. 

## 7. Two-Dimensional Fractionation

Orthogonal separations prior to MS analysis, such as strong cation exchange (SCX) fractionation and reverse phase (high pH)-reverse phase (low pH) extraction achieved low ng/mL LOQ with acceptable CVs (coefficient of variations) [51, 52]. These approaches may be useful to prioritize targets when we deal with tens of different targets at the same time. These separations usually utilize immunodepletion of high-abundant proteins along with extensive 2-dimentional (2-D) fractionation. Although it allows us to quantify low-abundant protein targets (~ng/mL), it may have some caveats, such as reducing overall throughput, increasing assay cost, causing potential false positives due to the complex sample processing, and causing potential false negatives due to the removal of interesting targets along with the high abundant proteins. Unlike the other techniques mentioned in Sections 2 to 6, sample preparation is not easily automated.

The combination of strong cation exchange and reverse phase chromatography (SCX-RP) is a well-known 2-D peptide separation technique used to separate peptides in complex samples. This combination can be used as online (e.g., MudPIT; multidimensional protein identification technology) or offline SCX-RP. In addition to its role in reducing sample complexity, the advantage of SCX-RP is its orthogonal separation of peptides using different biochemical properties, such as charge states of the peptides, which make it possible to identify low-abundance proteins. In addition, the application of SILAC-MRM (MRM of stable isotope labeling by/with amino acids in cell culture) or mTRAQ-MRM (MRM of mTRAQ-labeled peptides) technology increased the abilities of SCX-RP [53, 54]. For instance, Shah et al. successfully quantified the fifteen-candidate biomarkers in human cervicovaginal fluid (CVF) samples from term and preterm birth (PTB) cases [54], and DeSouza et al. applied SCX-RP with the mTRAQ-MRM technology to quantify two endometrial cancer biomarkers: pyruvate kinase (PK) and polymeric immunoglobulin receptor (PIGR) [53]. The concentration range of PK and PIGR was from less than 5 pmol/mg to several hundreds pmol/mg. 

**Figure 3:**
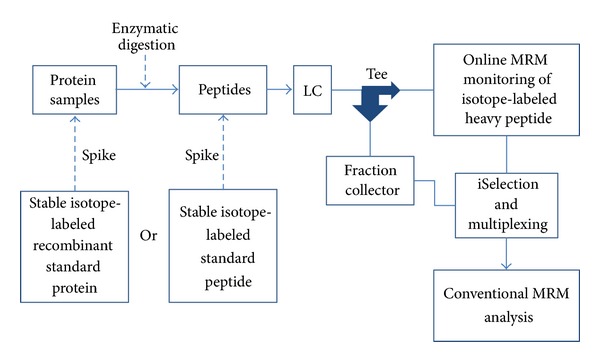
Schematic view of high-pressure, high-resolution separations coupled with intelligent selection and multiplexing (PRISM).

 Very recently, a new antibody-free technology, known as high-pressure, high-resolution separations coupled with intelligent selection and multiplexing (PRISM), used to detect low-abundant proteins in biofluids was developed by Shi et al. (Figure 3) [55]. The robustness of this technology is due to the online SRM monitoring of the heavy isotope-labeled synthetic peptide internal standards during the first-dimensional separation, which was performed by a reversed-phase liquid chromatographic enrichment step in the pH 10 mobile phase. Using a tee union, they separated the flow streams 1 : 10. While the major eluents were collected every minute in 96 well plates, a small amount of the column eluents went to the mass spectrometer for online SRM monitoring of spiked peptides. With the advantage of selection power, which they called intelligent selection or iSelection, they could reduce the number of fractions to be analyzed in the next step, and also the fractions of interest were easily multiplexed with other target peptide fractions. To evaluate the sensitivity of this assay, they spiked prostate-specific antigen (PSA), which was the first FDA-approved prostate cancer marker for early detection of cancer in blood, into human female serum and measured it accurately and reproducibly in the 50–100 pg/mL range.

SDS-PAGE-based protein separation is one of the most popular methods in the field of protein biochemistry. Some of the researchers found that this traditional technique could be useful in targeted MRM analysis [56, 57]. 1-dimentional (1-D) SDS-Gel/MRM assay is a powerful but simple approach for targeted analysis. Samples are separated using 1-D SDS-PAGE. The protein samples from 1-D SDS-PAGE are well fractioned and can be easily used to directly target certain molecular weight proteins. In addition, 1-D SDS-PAGE can be used simply for enrichment purposes without using antibodies [57]. Researchers can easily get specifically sized protein samples from the gel slices. With this technology, Halvey et al. quantified tumor-derived mutant KRAS (v-Ki-ras-2 Kirsten rat sarcoma viral oncogene) oncoprotein in fluid from benign pancreatic cysts and pancreatic cancers at concentrations from 0.08 to 1.1 fmol/*μ*g protein [57], and Ang and Nice detected colorectal cancer-associated proteins (CCAPs) in the feces from a patient with colorectal cancer [56].

## 8. Concluding Remarks

While pharmaceuticals move toward the personalized medicine concept in many disease areas, the development of new biomarkers and diagnostics are essential for this realization. Patient stratification to show a favorable treatment response and monitoring the drug efficacy can be completed with suitable biomarkers. To select real biomarker(s) from the large number of candidates, it is widely acknowledged that optimal validation tools are required [58]. Many promising target protein quantification tools are available, and each platform has its own advantages and challenges (Table 1). They have complementary roles in the validation process, especially when one approach encounters a challenge. The first step of the validation process would be analytical verification. If we categorize a long list of candidates by the decision tree, it may be easier to move forward to find clinically useful biomarkers. 

## Figures and Tables

**Table 1:** The advantages, challenges, and applications of each target protein quantification technique.

	Advantages	Challenges	Applications
SRM/MRM-based assays	(i) High selectivity (ii) Low development cost (iii) Can be developed rapidly (iv) Multiplex possibility (v) Distinguish close related targets (vi) Measure low or nonimmunogenic targets	(i) Low sensitivity (ii) Not easy to standardize (multiple efforts in progress) (iii) Complex sample preparation (iv) High instrument cost (v) Relatively low throughput	(i) Relatively high abundant targets without antibody for initial analytical validation (ii) Small peptide targets (iii) Low or nonimmunogenic targets
ELISA, immunoassays	(i) High sensitivity (ii) Low operating cost (iii) No or simple sample preparation (iv) Easy to standardize and distribute (v) High throughput (vi) Automation	(i) Development cost is high (ii) Long timeframe to acquire a good antibody (iii) Hook-effects (iv) Highly challenging on PTM (v) Very hard to deal with low-immunogenic target (vi) Cross-reactivity	(i) Clinical applications when good reagents are available (ii) Low abundant targets
Immuno-mass spectrometry	(i) High selectivity (ii) High sensitivity (iii) Multiplex possibility (iv) Distinguish close related targets	(i) Still requires at least one high-affinity antibody and an expensive instrumentation	(i) Low abundant target and at least one high affinity antibody available (ii) Undistinguishable by antibody

## Abbreviations

SRM: Selected reaction monitoring
SISCAPA: Stable Isotope Standards and Capture by Anti-Peptide Antibodies
MSIA: Mass spectrometry immunoassay
IMAC: Immobilized metal affinity chromatography.
